# Prediction of Lower Grade Insular Glioma Molecular Pathology Using Diffusion Tensor Imaging Metric-Based Histogram Parameters

**DOI:** 10.3389/fonc.2021.627202

**Published:** 2021-03-10

**Authors:** Zhenxing Huang, Changyu Lu, Gen Li, Zhenye Li, Shengjun Sun, Yazhuo Zhang, Zonggang Hou, Jian Xie

**Affiliations:** ^1^ Department of Neurosurgery, Beijing Tiantan Hospital, Capital Medical University, Beijing, China; ^2^ National Clinical Research Center for Neurological Diseases (China), Beijing, China; ^3^ Department of Neurosurgery, Peking University International Hospital, Beijing, China; ^4^ Neuroimaging Center, Beijing Neurosurgical Institute, Capital Medical University, Beijing, China; ^5^ Beijing Neurosurgical Institute, Capital Medical University, Beijing, China

**Keywords:** DTI metrics, insula glioma, molecular pathology prediction, simplified lesion drawing, histogram analysis

## Abstract

**Objectives:**

To explore whether a simplified lesion delineation method and a set of diffusion tensor imaging (DTI) metric-based histogram parameters (mean, 25th percentile, 75th percentile, skewness, and kurtosis) are efficient at predicting the molecular pathology status (MGMT methylation, IDH mutation, TERT promoter mutation, and 1p19q codeletion) of lower grade insular gliomas (grades II and III).

**Methods:**

40 lower grade insular glioma patients in two medical centers underwent preoperative DTI scanning. For each patient, the entire abnormal area in their b-non (b0) image was defined as region of interest (ROI), and a set of histogram parameters were calculated for two DTI metrics, fractional anisotropy (FA) and mean diffusivity (MD). Then, we compared how these DTI metrics varied according to molecular pathology and glioma grade, with their predictive performance individually and jointly assessed using receiver operating characteristic curves. The reliability of the combined prediction was evaluated by the calibration curve and Hosmer and Lemeshow test.

**Results:**

The mean, 25th percentile, and 75th percentile of FA were associated with glioma grade, while the mean, 25th percentile, 75th percentile, and skewness of both FA and MD predicted IDH mutation. The mean, 25th percentile, and 75th percentile of FA, and all MD histogram parameters significantly distinguished TERT promoter status. Similarly, all MD histogram parameters were associated with 1p19q status. However, none of the parameters analyzed for either metric successfully predicted MGMT methylation. The 25th percentile of FA yielded the highest prediction efficiency for glioma grade, IDH mutation, and TERT promoter mutation, while the 75th percentile of MD gave the best prediction of 1p19q codeletion. The combined prediction could enhance the discrimination of grading, IDH and TERT mutation, and also with a good fitness.

**Conclusions:**

Overall, more invasive gliomas showed higher FA and lower MD values. The simplified ROI delineation method presented here based on the combination of appropriate histogram parameters yielded a more practical and efficient approach to predicting molecular pathology in lower grade insular gliomas. This approach could help clinicians to determine the extent of tumor resection required and reduce complications, enabling more precise treatment of insular gliomas in combination with radiotherapy and chemotherapy.

## Introduction

Gliomas are a highly infiltrative form of neoplasm that remain challenging to treat. In recent years, the identification of molecular alterations (including IDH, TERT promoter mutation, MGMT methylation, and 1p/19q codeletion) associated with the prognosis of glioma. Furthermore, it has also diminished differences in outcome between grade II and III gliomas (so-called lower grade gliomas) that share the same molecular subtype (1). Due to the lower invasiveness and sensitivity to chemoradiotherapy associated with this specific molecular subtype, lower grade gliomas have been reported to have a relatively better survival time, with a range of 1–15 years (2).

The insula is believed to be a preferential region for glioma formation, accounting for 25% of low-grade gliomas and 10.8% of GBMs in the supratentorial area (3). However, the insula is located deep within the Sylvian fissure and is covered by the M2 segment of the middle cerebral artery, which means that tumor resection can only be performed between this arterial network. Moreover, the lenticulostriate arteries are usually affected by insular gliomas, and should be carefully identified and preserved during tumor resection. All of these considerations mean that it is particularly important during insular glioma surgery to achieve maximal resection of the tumor while preserving function, which also contributes to enhancing the survival time and quality of life of the patient (4, 5). This surgery needs to be carried out well especially for lower grade insular gliomas, which are less invasive compared with GBMs. A more aggressive resection of the insular area can lead to refractory hemiplegia, aphasia, and a reduced quality of life, or even cause a severe and life-threatening delayed hematoma. As mentioned above, the pattern of molecular alterations in a glioma is highly associated with their biological behavior, sensitivity to chemoradiotherapy, and prognosis (6–8). Thus, a tumor near vital blood vessels could appropriately be considered residual when a preoperative prediction of a better prognosis was made based on its molecular pathology. Along with this perspective, formulating an accurate and practical method for predicting the molecular subtype of a tumor preoperatively is essential, but this remains challenging.

Attempting to predict molecular alterations using MRI scanning has been widely accepted as a rational approach to glioma treatment, and as such it has become an inevitable part of the treatment process. Due to the abundant nerve fiber projections present in insular gliomas (9), diffusion tensor imaging (DTI)-based tractography is valuable during surgical planning and has been found to preserve function (10); therefore, it has been applied preoperatively in many centers (11–13). Two DTI metrics, fractional anisotropy (FA) and mean diffusivity (MD), have been widely used in the prediction of glioma characteristics (14–16), and have been reported to be sufficient for predicting molecular alterations relative to the use of more advanced diffusion imaging methods (17). Typically, in most of these studies, the tumor core is defined as the region of interest (ROI), which should exclude the edema, cyst, necrotic region, and so on (18). However, it should be noted that the delineation of the tumor core is difficult and subjective due to the dependence of this method on the level of experience of the researcher performing the delineation (19). Additionally, the edema, cyst, or necrotic region also reflect tumor characteristics, and these regions might not be successfully excluded. Moreover, the procedure involved in aligning among different MRI modalities, which is used to locate the edema or the enhancing areas, can also lead to somewhat of a bias in the delineation and are time consuming. All of these factors limit the clinical application of this approach.

Therefore, in this study, we aimed to explore a more practical approach to predicting molecular alterations in lower-grade insular gliomas using a simplified method for defining the ROI. This was based on identifying the whole abnormal area of the b-non (b0) image from the DTI scan sharing a high T2 weight that fit the original FA and MD maps without being transformed. In order to obtain more convincing results, we recruited patients from two medical centers with different MR scanners and DTI protocols. Then, based on different histogram parameters of FA, MD and their combination, we investigate the efficiency of the simplified ROI definition method in the prediction of lower-grade insular glioma molecular subtypes.

## Materials and Methods

### Study Population

A total of 40 insular glioma patients were recruited from Beijing Tiantan Hospital and Peking University International Hospital. The postoperative pathology examination proved that the tumor was a lower-grade glioma (grade II and III according to the WHO 2016 classification). All the patients were newly diagnosed with unilateral insular gliomas and no other intracranial lesions. Patient demographic and clinical data were retrieved from the medical records. The present study was approved by the Institutional Review Board of Beijing Tiantan Hospital and Peking University International Hospital.

### MRI Acquisition

Thirty-two patients were scanned using a Siemens Prisma 3.0 T scanner in Beijing Tiantan Hospital. Echo planar imaging (EPI) was utilized for DTI scanning, with the following parameters: diffusion-encoding directions = 30; b-values = 1,000 and 0 s/mm^2^; FOV = 256 mm; TE = 91 ms; TR = 10,000 ms; and slice thickness = 2 mm. The DTI scanning duration was approximately 17 min.

The remaining eight patients underwent DTI scanning in a Siemens Verio 3.0 T scanner in Peking University International Hospital. The scanning protocol used the following parameters: diffusion-encoding directions = 30; b values = 1,000 and 0 s/mm^2^; FOV = 230 mm, TE = 95 ms; TR = 3,600 ms; and slice thickness, 4 mm. The DTI scanning duration was approximately 9 min.

Conventional MR sequences, including T1-weighted images, T2-weighted images, fluid-attenuated inversion recovery (FLAIR) images, and T1-weighted images with intravenous injection of a gadolinium contrast agent were used to assess all patients during preliminary treatment planning.

### DTI Image Processing

DTI images were processed using FSL 6.0 software (http://www.fmrib.ox.ac.uk/fsl). First, the Brain Extraction Tool (BET) was used to extract the brain tissue portion of the b0 image (20). Then, an eddy-current correction was applied to adjust for the effects of the gradient coils on the DTI images. Finally, the DTIFIT toolbox (21) was used to generate the FA and MD maps.

### ROI Definition and Histogram Parameter Extraction

After BET processing, the b0 images were used to generate an ROI from a binary image mask using the Medical Imaging Interaction Toolkit (MITK) (http://www.mitk.org). The delineation was performed by a neurosurgeon with eight years of experience, with a hyperintensity on the b0 BET image defined as the ROI. As the b0 images were part of the whole DTI image series, it was possible to align the ROI well with the FA and MD maps.

For the histogram parameters, we extracted the mean, 25th percentile, and 75th percentile values of the voxels in the ROI. Moreover, the values of every individual voxel in the ROI were also extracted to calculate the skewness (asymmetry) and kurtosis (peak frequency) of the histogram, reflecting the characteristics of the histogram distribution. Fslstats (part of the FSL software package) was used to extract the voxel values.

### Group Criteria and Statistical Analysis

According to the results of the pathological examination, all the patients were assigned to one of each of the following pairs of groups: glioma grade 2 or grade 3; MGMT-methylated or MGMT-unmethylated; IDH-mutated (IDH-mu) or IDH-wildtype (IDH-wt); TERT promoter-mutated (TERTp-mu) or TERT promoter wildtype (TERTp-wt); and 1p19q codeletion or non-1p19q codeletion.

The Kolmogorov–Smirnov test was performed to confirm the Gaussian distribution of all continuous variables, with only the kurtosis of MD having a non-normal distribution. Then either a Mann–Whitney test or an independent t-test was performed to compare histogram parameters and age differences between groups. In order to illustrate the changing trend between DTI metrics and groups, we plotted the heatmap for p values that generated in comparisons mentioned above and performed Pearson correlation analyses for different DTI metrics. For statistically significant DTI metrics, a receiver operating characteristic (ROC) curve analysis was also used to evaluate the diagnostic efficiency individually and jointly, from which the area under the curve (AUC) and cutoff value were also acquired. Finally, we evaluated the reliability of DTI metrics in predicting molecular pathology *via* calibration curve. Considering our relatively small samples, an internal validation with 1000 of bootstrap replicates of original dataset was used. The Harrell’s concordance index (C-index) was believed to be an indicator for evaluating the discrimination, which was numerically equal to the AUC. Moreover, we also calculated the corrected C-index that generated after 1000 runs of bootstrap replication. The adequacy of these predictions was also evaluated by and Hosmer and Lemeshow (HL) test, in which the statistical insignificance (*P* > 0.05) implies a goodness of fit in the prediction.

SPSS 26.0, MATLAB 2017b, GraphPad Prism 8, and R studio were used to perform the statistical analyses and to generate statistical plots. A *P*-value < 0.05 on a two-tailed test was considered statistically significant.

## Results

### Demographic Characteristic

Detailed demographic characteristics are shown in **Table 1**.

**Table 1:** Demographic characteristic.

Variables	Tumor grade and molecular pathology status									
	Grade2	Grade3	MGMT methylated	Non-MGMT unmethylated	IDH-mu	IDH-wt	TERTp-mu	TERTp-wt	1p/19q-codel	Non 1p19q-codel
Total No.	26	14	30	10	32	8	16	24	14	26
Gender, M/F, n	17/9	9/5	21/9	5/5	21/11	5/3	12/4	14/10	9/5	17/9
Age (Mean ± SD), year	40.4 ± 11.8	45.9 ± 7.5	43.8 ± 10.1	37.7 ± 11.8	40.6 ± 10.7	49.0 ± 8.4	45.9 ± 9.6	40.0 ± 11.0	43.9 ± 13.3	41.4 ± 9.3

### Histogram Parameter Comparisons and Prediction Efficiency

#### Histogram Overlay

**Figure 1:**
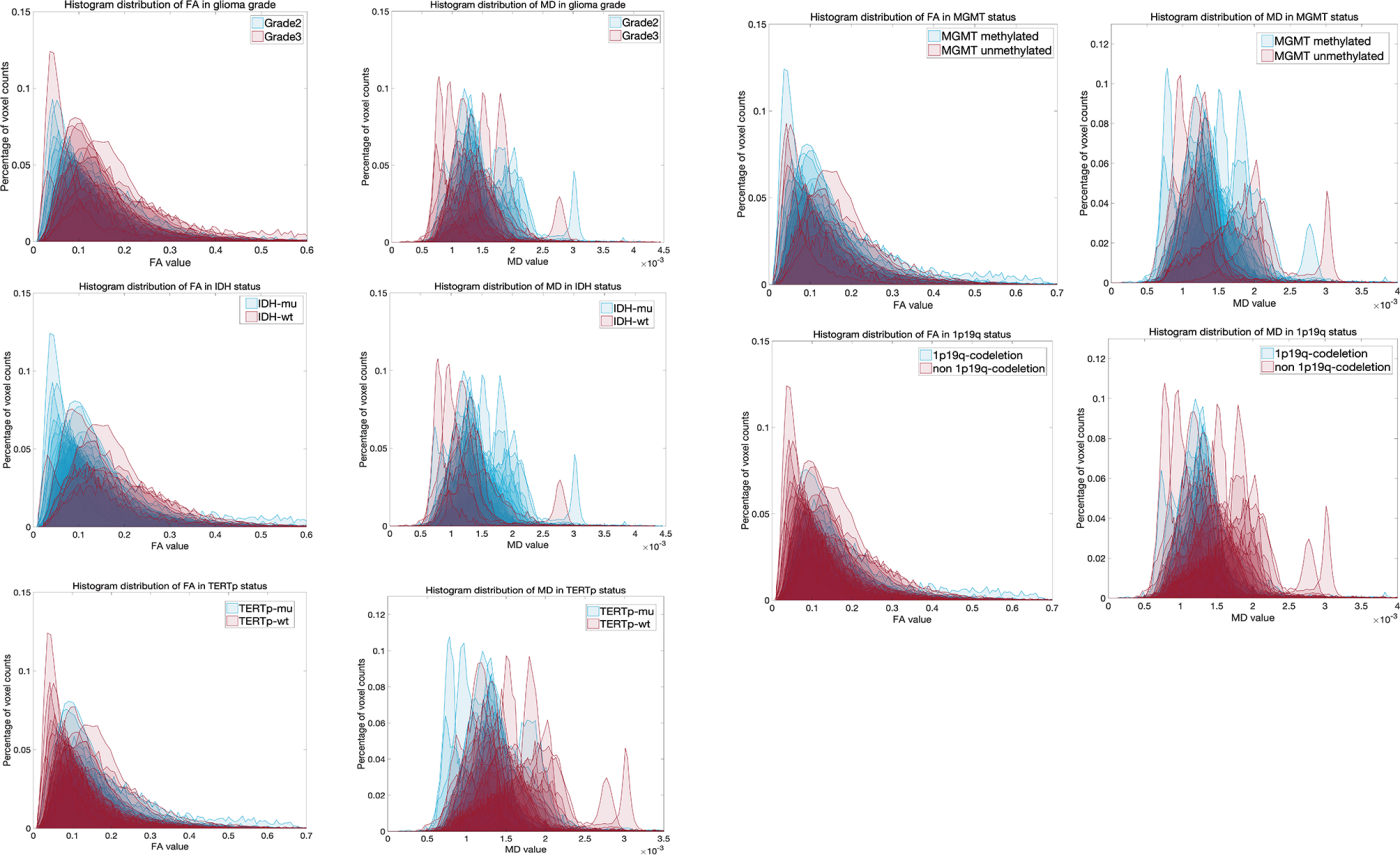
Individual histogram overlay for grade and molecula pathology status.

The histogram overlay for patients from different groups is shown in **Figure 1**. From this figure, it is possible to develop a preliminary understanding of the differences in histogram distributions between data from patients with different glioma grade or molecular pathology status.

#### Grade 2 vs. Grade 3

**Figure 2:**
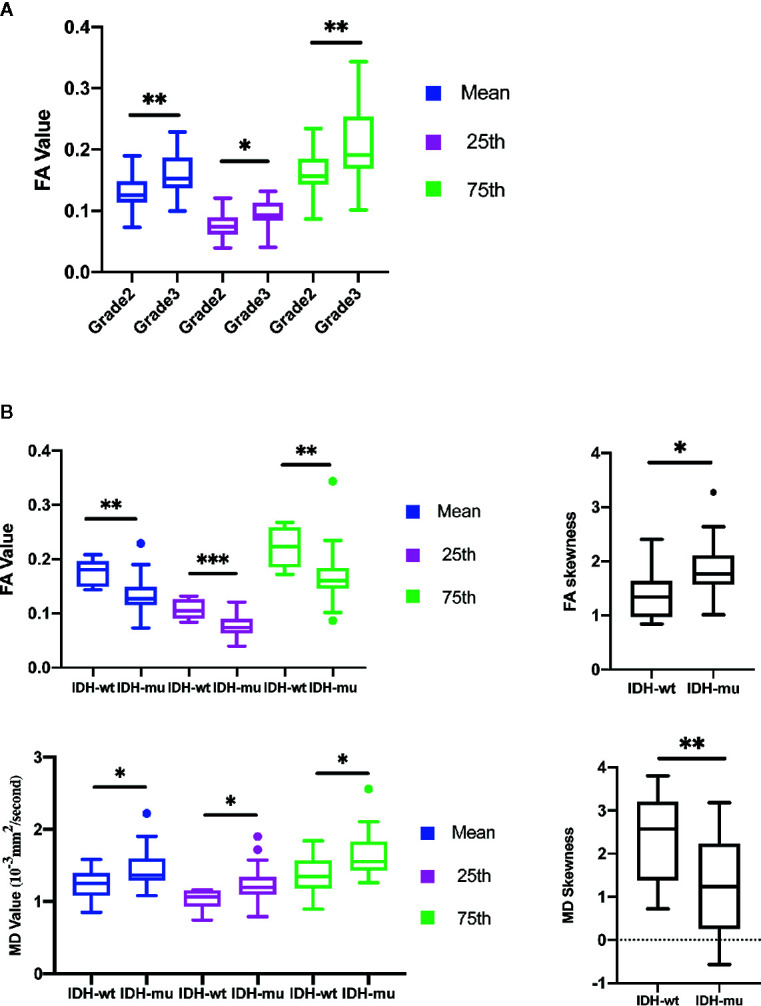
Histogram parameters comparison in group of Grades **(A)** and IDH mutation status **(B)**. Only statistically significant parameters were shown. *P < 0.05, **P < 0.01.

The mean, 25th percentile, and 75th percentile of FA showed statistically significant differences, with grade 3 insular gliomas having higher values for a number of different FA histogram parameters (**Table 2**, **Figures 2A** and **5A**). However, the 25th percentile of FA exhibited the highest AUC (0.750) as separate indicator, while their combination showed the best discrimination (AUC 0.772) in terms of distinguishing the tumor grade. More detailed information is shown in **Table 3** and **Figure 4A**.

**Table 2:** Histogram parameters comparisons between different grade and molecular pathology status.

DTI parameters	Tumor grade and molecular pathology status														
	Grade2	Grade3	P	MGMT methylated	MGMT unmethylated	P	IDH-mu	IDH-wt	P	TERTp-mu	TERTp-wt	P	1p/19q-codel	Non 1p19q-codel	P
**FA**															
Mean	0.131 ± 0.027	0.162 ± 0.036	0.004	0.14 ± 0.032	0.147 ± 0.04	0.602	0.133 ± 0.031	0.175 ± 0.024	0.001	0.161 ± 0.034	0.129 ± 0.027	0.002	0.152 ± 0.034	0.136 ± 0.033	0.150
25th	0.076 ± 0.018	0.094 ± 0.024	0.011	0.082 ± 0.019	0.086 ± 0.031	0.661	0.077 ± 0.018	0.107 ± 0.019	<0.001	0.095 ± 0.02	0.074 ± 0.019	0.002	0.088 ± 0.016	0.08 ± 0.024	0.239
75th	0.162 ± 0.035	0.207 ± 0.061	0.021	0.176 ± 0.05	0.184 ± 0.051	0.655	0.167 ± 0.046	0.222 ± 0.037	0.003	0.204 ± 0.055	0.16 ± 0.037	0.004	0.193 ± 0.054	0.169 ± 0.046	0.151
Skewness	1.754 ± 0.387	1.681 ± 0.695	0.671	1.753 ± 0.538	1.656 ± 0.42	0.609	1.817 ± 0.476	1.375 ± 0.504	0.025	1.649 ± 0.537	1.782 ± 0.492	0.424	1.623 ± 0.4	1.786 ± 0.556	0.339
Kurtosis	4.577 ± 2.151	4.962 ± 4.038	0.695	4.842 ± 3.122	4.322 ± 2.218	0.630	5.04 ± 2.881	3.401 ± 2.789	0.156	4.446 ± 3.431	4.889 ± 2.558	0.642	3.88 ± 2.451	5.16 ± 3.072	0.187
**MD**															
Mean (10^-3^mm^2^/s)	1.445 ± 0.245	1.327 ± 0.229	0.145	1.393 ± 0.203	1.437 ± 0.349	0.715	1.445 ± 0.233	1.238 ± 0.223	0.029	1.293 ± 0.196	1.478 ± 0.247	0.017	1.283 ± 0.1	1.469 ± 0.273	0.004
25th (10^-3^mm^2^/s)	1.245 ± 0.22	1.112 ± 0.214	0.074	1.19 ± 0.2	1.221 ± 0.299	0.714	1.239 ± 0.223	1.033 ± 0.147	0.018	1.11 ± 0.195	1.257 ± 0.228	0.041	1.091 ± 0.118	1.256 ± 0.248	0.007
75th (10^-3^mm^2^/s)	1.619 ± 0.304	1.488 ± 0.275	0.185	1.555 ± 0.239	1.629 ± 0.44	0.621	1.626 ± 0.28	1.363 ± 0.287	0.023	1.427 ± 0.227	1.67 ± 0.303	0.009	1.417 ± 0.095	1.657 ± 0.335	0.002
Skewness	1.337 ± 1.192	1.618 ± 1.277	0.493	1.376 ± 1.126	1.613 ± 1.5	0.655	1.189 ± 1.133	2.42 ± 1.063	0.008	2.056 ± 1.063	1.022 ± 1.146	0.007	2.092 ± 0.622	1.082 ± 1.313	0.002
Kurtosis	6.028 (1.572-14.054)	4.985 (1.281- 16.152)	0.812	5.544 (1.494- 13.084)	11.434 (0.382-16.507)	0.612	4.909 (1.274-13.718)	13.338 (6.044-7.872)	0.082	12.531 (5.060-15.923)	2.175 (0.209-10.199)	0.008	12.045 (5.345-15.707)	2.082 (0.519-12.816)	0.019

**Table 3:** Diagnostic efficiency.

Parameter	Cutoff value	Sensitivity	Specificity	AUC (95% CI)
**Grade**				
FA mean	0.131	0.857	0.615	0.747 (0.584–0.911)
FA 25th	0.082	0.857	0.692	0.750 (0.585–0.915)
FA 75th	0.164	0.857	0.615	0.747 (0.579–0.915)
Combination	–	0.857	0.615	0.772(0.624–0.920)
**IDH mutation**				
FA mean	0.141	1	0.656	0.856 (0.735–0.976)
FA 25th	0.082	1	0.625	0.879 (0.763–0.994)
FA 75th	0.170	1	0.656	0.863 (0.747–0.980)
FA skewness	1.468	0.813	0.750	0.766 (0.551–0.981)
MD mean	1.271×10-3	0.844	0.625	0.746 (0.547–0.945)
MD 25th	1.170×10-3	0.563	1	0.789 (0.636–0.942)
MD 75th	1.376×10-3	0.906	0.625	0.762 (0.551–0.973)
MD skewness	2.179	0.75	0.75	0.801 (0.633–0.969)
Combination	–	0.781	1	0.930 (0.847–1.000)
**TERTp mutation**				
FA mean	0.137	0.813	0.708	0.794 (0.648–0.941)
FA 25th	0.080	0.875	0.708	0.794 (0.649–0.939)
FA 75th	0.168	0.813	0.708	0.779 (0.628–0.929)
MD mean	1.292×10-3	0.833	0.562	0.727 (0.567–0.886)
MD 25th	1.273×10-3	0.458	0.937	0.661 (0.490–0.833)
MD 75th	1.472×10-3	0.75	0.75	0.746 (0.590–0.902)
MD skewness	1.437	0.813	0.667	0.740 (0.581–0.898)
MD kurtosis	6.786	0.75	0.75	0.747 (0.595–0.899)
Combination	–	0.813	0.792	0.841 (0.717–0.966)
**1p19q codeletion**				
MD mean	1.336×10-3	0.214	0.786	0.773 (0.630–0.917)
MD 25th	1.252×10-3	0.5	1	0.714 (0.558–0.870)
MD 75th	1.448×10-3	0.808	0.786	0.783 (0.637–0.929)
MD skewness	0.910	1	0.615	0.739 (0.585–0.893)
MD kurtosis	3.257	1	0.577	0.725 (0.569–0.881)
Combination	–	1	0.615	0.772 (0.623–0.921)

The calibration curve exhibited prediction and observation data agreed fine in internal validation (**Figure 6A**), which also demonstrated by the HL test that revealed no significance (P = 0.424). And the corrected C-index was 0.702.

#### IDH-wt vs. IDH-mu

The mean, 25th percentile, 75th percentile, and skewness of FA and MD were sufficient to distinguish IDH mutation status (**Table 2** and **Figure 2B**). A higher mean, 25th percentile, and 75th percentile, and a lower skewness of FA were associated with IDH-wt, while the MD histogram parameters followed the opposite trend (**Figure 5A**). Moreover, the diagnostic efficiency among these parameters varied, with the 25th percentile of FA yielding the highest diagnostic efficiency with the AUC of 0.879. The efficiency of the combination among these parameters could reach to 0.930. More detailed information is shown in **Table 3** and **Figure 4B**.

The calibration curve of IDH prediction also showed the corrected curve agreed fine with ideal reference line in internal validation (**Figure 6B**). The HL test also exhibited an insignificance with P value of 0.434. And the corrected C-index was 0.793.

#### TERTp-wt vs. TERTp-mu

**Figure 3:**
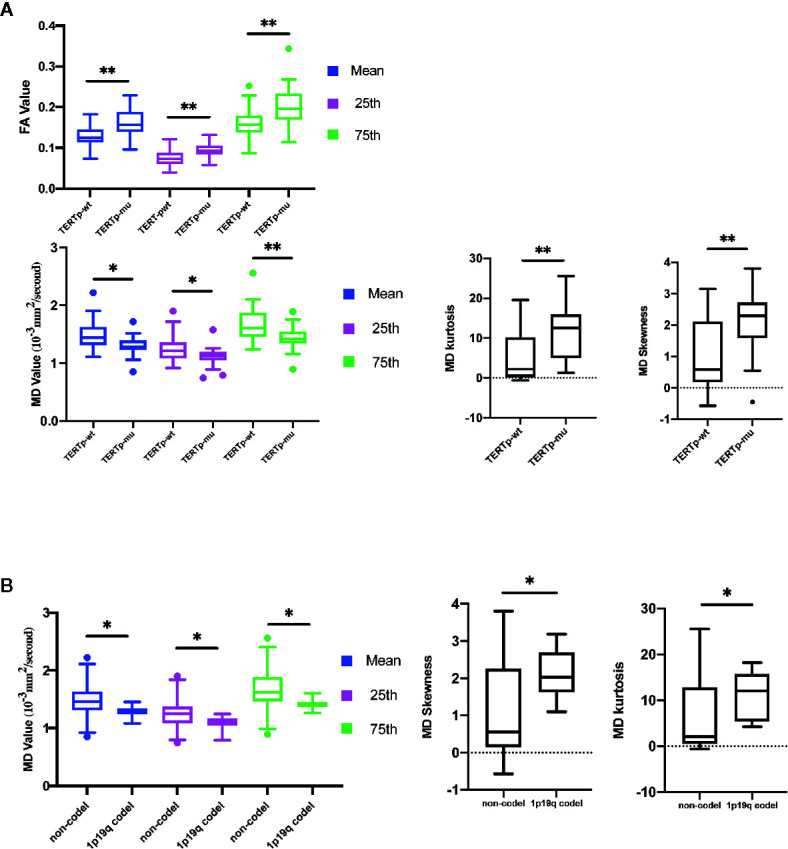
Histogram parameters comparison in group of TERT promoter mutation **(A)** and 1p19q codeletion status **(B)**. Only statistically significant parameters were shown. *P < 0-05, **P < 0.01.

**Figure 4:**
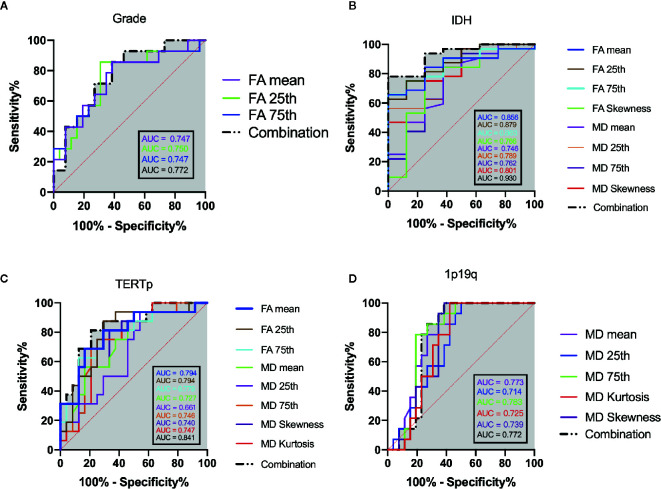
ROC analysis of the statistically significant histogram parameters of FA, MD and their combination when patients were stratified as: **(A)** grade, **(B)** IDH mutation, **(C)** TERT promoter mutation. **(D)** 1p19q codeletion. The grey shadow stand for the AUC of combination.

**Figure 5:**
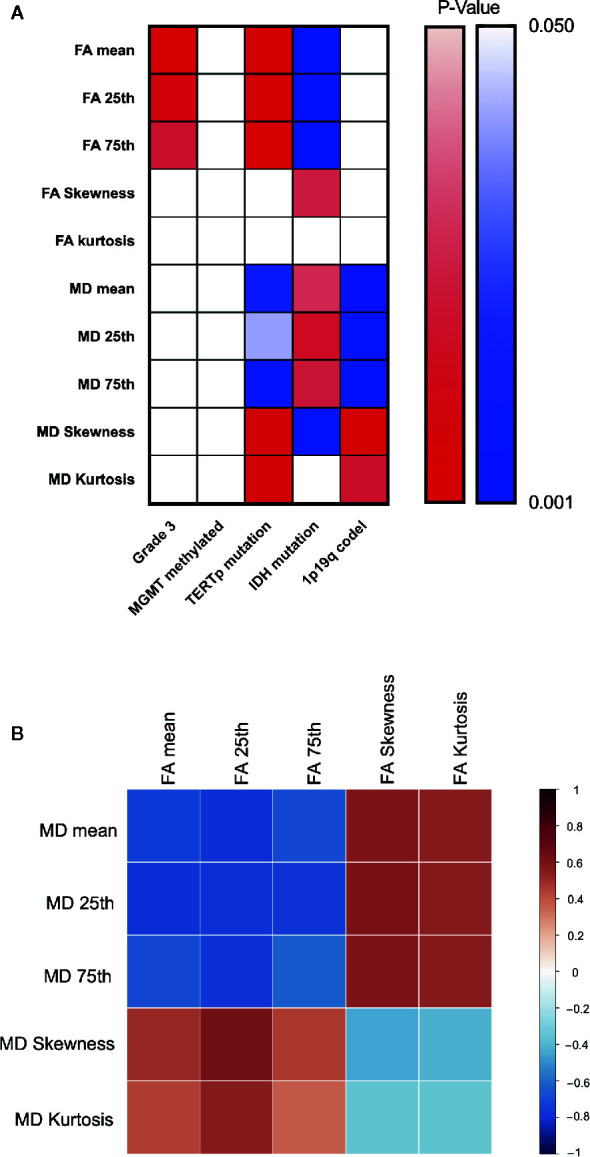
Heatmap for changing trend between different indexes. P value plotting **(A)** between molecular pathology and DTI metrics. Warm colour theme implied positive changing trend between each labels, cool colour theme meant the negative one. White colour stand for statistically insignificance. Pearson correlation analysis between DTI metrics **(B)**.

The mean, 25th percentile, and 75th percentile of FA were associated with TERT promoter mutation. Furthermore, all MD histogram parameters exhibited diagnostic value at predicting TERT promoter mutation (**Table 2** and **Figure 3A**). The overall trend of these parameters showed that mutation of the TERT promoter was associated with elevated FA values and decreased MD values. In addition, higher skewness and kurtosis of the MD histogram were also associated with TERT promoter mutation (**Figure 5A**). In terms of prediction efficiency, the mean and 25th percentile of FA shared the same AUC; however, the latter parameter exhibited a higher sensitivity. And the combination of significant FA and MD parameters showed the highest AUC to predict TERT promoter mutation, with the AUC of 0.841. Detailed information is shown in **Table 3** and **Figure 4C**.

**Figure 6:**
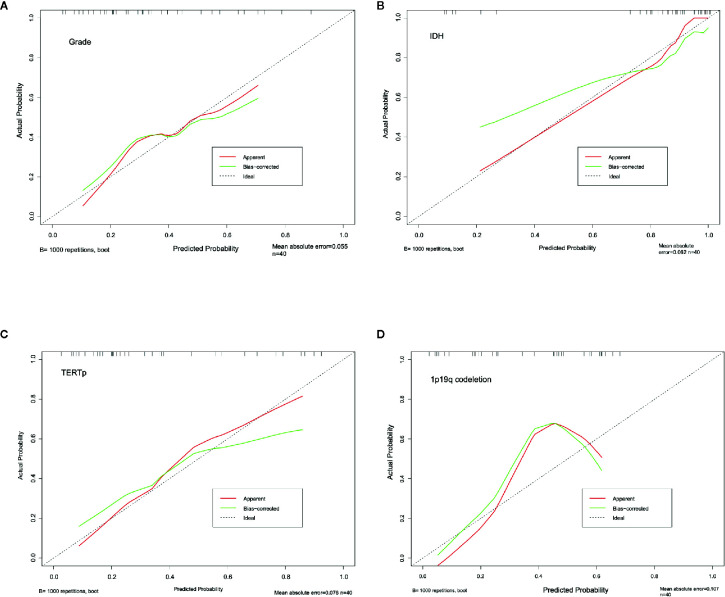
Calibration curve for grading **(A)**, IDH **(B)**, TERTp **(C)** and 1p19q codeletion **(D)**. The dash line stand for the ideal prediction, which the predicted probability equal to actural probability. And red line stand for the apparent prediction performance, the green line indicade the bootstrap method corrected prediction performance.

The calibration curve of TERTp prediction exhibited an agreement with ideal reference line in internal validation (**Figure 6C**), which also corresponded with insignificance with P value of 0.338 in HL test. And the corrected C-index was 0.705.

#### Non-1p19q Codeletion vs. 1p19q Codeletion

None of the FA histogram parameters showed statistically significant prediction of 1p19q codeletion, while all of the MD parameters were significant (**Table 2**, **Figures 3B** and **5A**), with the 75th percentile of MD exhibiting the highest AUC of 0.783 (**Table 3** and **Figure 4D**).

However, the calibration curve of 1p19q codeletion prediction showed an unsatisfied corrected curve with ideal reference line in internal validation (**Figure 6D**). And the HL test exhibited a lowest P value of 0.173 when compared to other groups. And the corrected C-index of 1p19q prediction was the lowest, with the value of 0.675.

#### MGMT-Methylated vs. MGMT-Unmethylated

None of the FA- or MD-associated histogram parameters predicted MGMT methylation at *P* < 0.05. Detailed information of these analyses can be found in **Table 2**.

## Changing Trend Between Variables

Correlation heatmaps are shown in **Figure 5B**. Overall, we observed a trend in the changes of FA and MD values across groups, with higher FA values for grade 3, TERTp-mu while lower FA value for IDH-mu, and higher MD values for IDH-mu while lower MD value for TERTp-mu and 1p/19q codeletion. Moreover, the Pearson correlation further proved the negative changing trend between FA and MD values.

## Discussion

The results of our study demonstrate that the use of DTI metrics to delineate the entire abnormal region of b0 images was a valuable and simpler approach to discriminating the molecular subtype of lower grade insular gliomas. Furthermore, our results revealed a trend in which molecular subtypes associated with IDH and TERT promoter mutation status linked to a worse prognosis had higher FA values, but lower MD values. However, MD values were higher in 1p19q-intact patients, who are more likely to have a poorer prognosis. The skewness and kurtosis of the histograms exhibited the opposite trend compared with the mean or percentile values. Moreover, different histogram parameters were associated with different efficiencies at predicting molecular pathology. And a novel discrimination of grading, IDH and TERT mutation was observed under their combination.

The FA and MD calculated from three directional eigenvalues during DTI scanning reflect the directional preference of water molecule diffusion and the mean diffusion irrespective of its direction, respectively (22, 23). Thus, FA and MD both evaluate properties related to water diffusion, but from opposing perspectives. As white matter restricts water diffusion along its myelin sheath, white matter preservation has been shown to yield increased FA values, but decreased MD values (24), which has provided opportunities for diagnosing diseases affecting white matter, such as multiple sclerosis (25).

These DTI metrics have also been adopted for glioma diagnosis, with alterations to these DTI metrics in different grades or molecular subtypes of glioma believed to be triggered by the density of tumor cells. Beppu et al. (26) observed a positive correlation between FA and tumor cell density, while Zhao et al. (27) reported that MD was negatively correlated with Ki67, which is well known as an index of tumor cell proliferation. Similar evidence also indicates that tumor cell density, which depends on proliferative activity, is positively correlated with FA, but negatively correlated with MD (28, 29). Of the different tumor types, an aggressive glioma would be expected to lead to reduced cell organization and higher cell density, thereby restricting water molecule diffusion and further increasing FA but decreasing MD.

Based on this perspective, studies have reported characteristics of glioma aggressiveness, such as grading and molecular subtypes, have been associated with DTI metrics. In terms of glioma grading, Lee et al. (30) reported that MD, but not FA, values are lower in high-grade gliomas, while Inoue et al. and Zhao et al. (14, 27, 31) found complementary evidence that increased FA values are also observed in high-grade glioma. And Server et al. found an increasing trend in grade III glioma when compared to grade II (15). In the aspect of the molecular subtype, Aliotta et al. (32) found that mutation of IDH resulted in decreased FA values, while codeletion of 1p19q resulted in increasing FA trend, but not statistically significant. Park et al. (33) reported that FA was lower in glioma patients with IDH mutation and higher in those with 1p19q codeletion. Moreover, Figini et al. (34) found lower FA and higher MD values in glioma patients with IDH mutations; however, there were no significant result regarding the prediction of 1p19q codeletion.

Taken together, as we summarized in **Table 4**, the higher FA and lower MD we observed in IDH-wildtype and high-grade glioma are consistent with previous studies. Additionally, we also found this changing trend in 1p19q codeletion and TERT mutation gliomas, which seldom reported.

**Table 4:** Fractional anisotropy (FA) and mean diffusivity (MD) involved in aggressive assessment of glioma.

References	FA	MD	ROI
Beppu et al. (26) N = 19	Positive with tumor cell density	–	Enhancing area, avoiding necrosis
Zhao et al. (27) N = 52	Increasing in high grade (AUC: 0.74), IDH wildtype glioma (AUC: 0.67)	Decreasing in IDH wildtype glioma (AUC:0.67) and negative with Ki-67.	Tumor core, avoiding necrosis and cystic.
Tonoyan et al. (28) N = 47	Positive with glioma proliferative activity	Negative with glioma proliferative activity	Most malignant tumor part
Kinoshita et al. (29) N = 20	Positive with tumor cell density and Ki-67	Negative with tumor cell density and Ki-67.	Tumor core (Target for biopsy)
Lee et al. (30) N = 27	No significance was found in glioma grading	Decreasing in high grade glioma	non-enhancing area
Inoue et al. (14) N = 41	Increasing in high grade glioma	Decreasing in high grade glioma	Solid portion of tumor
Liu et al. (31) N = 52	Increasing in high grade glioma (AUC:0.928)	–	Solid tumor part
Server et al. (15) N = 78	FA of Grade 3 glioma was higher than Grade 2 glioma, but not statistically significant.	–	Solid tumor part
Aliota et al. (32) N = 41	Increasing in IDH wildtype gliomas (AUC: 0.90, when combined with ADC) An increasing trend in 1p19q codeletion gliomas, but not statistically significant.	–	The combination of automated segmentation of edema, contrast-enhancing area, and non-enhancing area.
Park et al. (33) N = 93	Increasing in IDH wildtype gliomas (AUC: 0.853, when combined other parameters)	–	Manually drawn on T2-weighted image.
Figini et al. (34) N = 192	Increasing in IDH wildtype gliomas (AUC: 0.74)	Decreasing in IDH wildtype gliomas (AUC:0.73)	Solid part of tumor

The link of IDH mutation with FA and MD might be attributed to gliomas with IDH mutations exhibiting more homogeneous tumor populations with lower cell densities (35). Mutation of the promoter of TERT, which encodes a telomerase, would lead to an uncontrolled proliferation of tumor cell and is believed to be a precondition for the formation of brain cancer (6); this could lead to higher FA and lower MD values, as we observed. Interestingly, the 1p19q codeletion group exhibited a decreased MD, which seems to conflict with the consistent results found with the other molecular subtypes, which showed better prognosis associated with lower FA and higher MD values. This result might be because 97% of 1p19q codeletion gliomas carry a TERT promoter mutation (2), leading to greater invasiveness, a trend that was also observed in our study. However, the 1p19q codeletion means that this type of gliomas are vulnerable to chemotherapy with an alkylating agent and exhibited a better prognosis after this treatment (8, 36). Lastly, MGMT, a DNA damage repair protein that removes guanine-alkyl groups and prevents apoptosis, has been shown to prevent the effect of temozolomide (37, 38). Methylation of MGMT inhibits the function of this protein, thereby make the tumor sensitive to temozolomide. Thus, MGMT methylation is distinct from the invasiveness of the glioma and was not related to alterations of FA or MD.

It should be noted that the method of ROI definition was not quite consistent among previous and our studies, however, the results were consistent, and the AUC were acceptable (all above 0.6). This phenomenon implied the FA and MD were strong enough to predict glioma grading or molecular subtypes, which smooth out the effect of different ROI delineation method. Thus, defining the entire abnormal area as the ROI could simplify the delineation procedure, which could improve the value of clinical use. Due to the lower grade gliomas were heterogeneous (39), with cystic or calcified regions and edema often evident. And the parameters with the highest prognostic efficiency were not the mean value of DTI metrics. These results provide evidence that under the whole abnormal area as ROI, the mean value of the ROI is not the best candidate for distinguishing the molecular subtype and grade of a glioma. This phenomenon is consistent with those of previous studies that have reported that the 25th percentile, 75th percentile, skewness, and kurtosis of the histogram can be used to improve the prediction of tumor characteristics (40, 41). Moreover, we also observed an enhanced discrimination of grade, IDH and TERT mutation when combined significant DTI metrics. And the calibration curve and HL test also yielded a fine fitness of prediction. Moreover, Aliota et al. (32) also defined all the abnormal area as ROI with an automated tumor segmentation method and abstracted different histogram value of FA and apparent diffusion coefficient (ADC) in ROI. The AUC of their optimal model to predict IDH mutation reached to 0.90, which was similar to our study (uncorrected AUC: 0.930, corrected AUC:0.793 for IDH mutation prediction).

We are aware of a number of limitations to this study. The first is the retrospective nature of the study and the relatively small sample size. And a larger dataset for external validation was still needed. The second is that, although we defined the ROI using a simplified method, this procedure was performed manually and is therefore still subjective. And the automated tumor segmentation *via* machine learning should be explored in future studies. In addition, we mainly focused on lower grade insular gliomas, and the question of whether this simplified ROI definition is applicable to gliomas in other brain areas still needs to be explored.

Overall, more invasive gliomas exhibited higher FA and lower MD values. A simplified ROI delineation procedure using the combination of appropriate histogram parameters yielded a more practical approach with efficiency in preoperatively predicting molecular alterations in lower grade insular gliomas. This could help to determine the extent of tumor resection and reduce complications following surgery, allowing for more precise treatment of insular gliomas in combination with radiotherapy and chemotherapy.

## Data Availability Statement

The original contributions presented in the study are included in the article/supplementary material. Further inquiries can be directed to the corresponding authors.

## Ethics Statement

The studies involving human participants were reviewed and approved by The Institutional Review Board of Beijing Tiantan Hospital and Peking University International Hospital. Written informed consent to participate in this study was provided by the participants’ legal guardian/next of kin.

## Author Contributions 

ZXH designed the study, analyzed the data, and drafted the manuscript. CL and JX provided the clinical and imaging data. GL and ZL performed the literature search. SS, YZ, ZGH, and JX reviewed and edited the manuscript. All authors contributed to the article and approved the submitted version.

## Funding

This study was supported by the National Natural Science Foundation of China (ID: 81771792) and the Beijing Municipal Science & Technology Commission (Z171100000117002).

## Conflict of Interest

The authors declare that the research was conducted in the absence of any commercial or financial relationships that could be construed as a potential conflict of interest.
